# Changes of Protein Expression after CRISPR/Cas9 Knockout of miRNA-142 in Cell Lines Derived from Diffuse Large B-Cell Lymphoma

**DOI:** 10.3390/cancers14205031

**Published:** 2022-10-14

**Authors:** Jennifer Menegatti, Jacqueline Nakel, Youli K. Stepanov, Karolina M. Caban, Nicole Ludwig, Ruth Nord, Thomas Pfitzner, Maryam Yazdani, Monika Vilimova, Tim Kehl, Hans-Peter Lenhof, Stephan E. Philipp, Eckart Meese, Thomas Fröhlich, Friedrich A. Grässer, Martin Hart

**Affiliations:** 1 Institute of Virology, Saarland University Medical School, 66421 Homburg, Germany; 2 Laboratory for Functional Genome Analysis (LAFUGA), Gene Center, Ludwig-Maximilians-University Munich, 81377 Munich, Germany; 3 Institute of Human Genetics, Saarland University, 66421 Homburg, Germany; 4 Center for Bioinformatics, Saarland University, 66041 Saarbrücken, Germany; 5 Experimental and Clinical Pharmacology and Toxicology, Saarland University Medical School, 66421 Homburg, Germany

**Keywords:** microRNA-142, CRISPR/Cas9, diffuse large B-cell lymphoma, miR-142 knockout cell lines, proteomics, transcriptomics, AKT1S1, CCNB1, LIMA1, TFRC

## Abstract

**Simple Summary:**

The gene of the human tumor suppressive microRNA-142 (miR-142) carries mutations in about 20% of cases of diffuse large B-cell lymphoma (DLBCL). Because microRNAs post-transcriptionally regulate the protein expression of their cognate messenger RNA (mRNAs) targets, we determined the effect of miR-142 knockout on protein expression in two cell lines derived from DLBCL. We found a significant up-regulation of 52 proteins but also a down-regulation of 41 proteins upon miR-142 deletion. Knockout of a miRNA may be used to identify novel targets, and seed-sequence mutants of a miRNA unable to bind to their targets can be used to confirm potential novel targets. With this approach, we identify AKT1S1, CCNB1, LIMA1 and TFRC as novel targets of miR-142. As miR-142 is highly present in the miRNA processing RISC complexes, the deletion of this miRNA might result in its replacement by other miRNAs, thus introducing an additional layer of complexity regarding gene regulation.

**Abstract:**

Background: As microRNA-142 (miR-142) is the only human microRNA gene where mutations have consistently been found in about 20% of all cases of diffuse large B-cell lymphoma (DLBCL), we wanted to determine the impact of miR-142 inactivation on protein expression of DLBCL cell lines. Methods: miR-142 was deleted by CRISPR/Cas9 knockout in cell lines from DLBCL. Results: By proteome analyses, miR-142 knockout resulted in a consistent up-regulation of 52 but also down-regulation of 41 proteins in GC-DLBCL lines BJAB and SUDHL4. Various mitochondrial ribosomal proteins were up-regulated in line with their pro-tumorigenic properties, while proteins necessary for MHC-I presentation were down-regulated in accordance with the finding that miR-142 knockout mice have a defective immune response. CFL2, CLIC4, STAU1, and TWF1 are known targets of miR-142, and we could additionally confirm AKT1S1, CCNB1, LIMA1, and TFRC as new targets of miR-142-3p or -5p. Conclusions: Seed-sequence mutants of miR-142 confirmed potential targets and novel targets of miRNAs can be identified in miRNA knockout cell lines. Due to the complex contribution of miRNAs within cellular regulatory networks, in particular when miRNAs highly present in RISC complexes are replaced by other miRNAs, primary effects on gene expression may be covered by secondary layers of regulation.

## 1. Introduction

To understand the contribution of genetic alterations in tumor formation, the concept of oncogenes and tumor suppressor genes has been developed [1]. Cell proliferation is activated via various mechanisms such as mutations, amplifications or translocations of oncogenes, while cell division may conversely be induced by deletion or mutational inactivation of growth suppressor genes. MicroRNAs (miRNAs), a class of small RNA molecules of 18–25 nt, are also known to have growth inducing or growth retarding effects [2]. MiRNAs are regulatory molecules that post-transcriptionally regulate gene expression and are functionally involved in a wide variety of biological processes (reviewed in [3]). They usually bind within the 3′ untranslated region (3′UTR) of their cognate target messenger RNA (mRNAs) and inhibit protein expression, either through reducing protein translation or by inducing target degradation [4]. Their role in cancer induction and maintenance by acting as proto-oncogenes or as tumor suppressor genes has been proposed [5] as miRNA genes are often located in genomic regions affected by alterations in cancer cells such as deletion(s) or overexpression/amplification (reviewed in [6] and references therein). This hypothesis was strengthened by the induction of leukemia in transgenic mice which constitutively express miR-155 from the Eµ-promoter [7]. In contrast, miR-34a is down-regulated in various tumors [8] and the miR-34a gene locus is deleted in neuroblastoma [9]. As a consequence, efforts are made to classify tumors, among other parameters, by their miRNA signature [10] or by the presence of secreted or released miRNAs in body fluids like blood or urine [11,12]. Some miRNAs appear to have either tumor-promoting or -suppressing functions depending on the type of tissue/cell where they are expressed [13].

DLBCL represent the majority of non-Hodgkin lymphoma (NHL) and are categorized either as germinal center B-cell-like (GCB), activated B-cell-like (ABC) or primary mediastinal large B-cell lymphoma (PMLBCL) [14]. During the miRNA profiling of primary DLBCL [15], we observed mutations of the miR-142 gene in approx. 20% of primary cases of DLBCL [16]. This finding was confirmed and extended to follicular lymphoma and also to acute myeloid leukemia (AML) [17,18,19,20]. More recently, it was shown that the mutation of miR-142-3p simultaneously resulted in reduced levels of miR-142-5p [20]. So far, no other mutations in the mature or the precursor molecules of other miRNAs have consistently been identified in lymphoma or leukemia, while sporadic somatic mutations in different miRNA genes in specimens of lung cancer have been observed [21]. The activation of c-MYC by juxtaposition of the miR-142 and the c-MYC locus have been described in IgκAID/p53−/− B-cell lymphomas [22,23]. MiR-142 is one of the miRNAs mentioned above [13] to exert tumor-suppressive or -promoting functions depending on the tissue where it is expressed. MiR-142 has tumor-suppressive properties, for instance in hepatocellular carcinoma [24] and reduced levels have also been found, among others, in colon [25], lung [26], or breast carcinoma [27]. Likewise, low levels of miR-142 and simultaneous high levels of miR-375 in serum confer a poor prognosis for patients with gastric carcinoma [28]. The report by Trissal et al. (2018) mentioned above [20] also pointed to a tumor-suppressive role of miR-142 as point mutations in the seed of miR-142-3p led to an increase in ASH1L, which in turn activates the expression of growth-promoting HOXA genes. High expression of miR-142 was also described for various leukemia, and high levels in AML appear to confer a positive prognosis [29]. In contrast, a tumor-promoting role for this miRNA has been proposed for prostate carcinoma [30]. Likewise, miR-142 appears to induce cell growth in adipose-derived stem cells [31].

The deletion of miR-142 in knockout mice resulted in a strongly deregulated lymphopoiesis and subsequent immunodeficiency [32,33]. Loss of function mutations in miR-142, as previously described by our group and others [16,17], was subsequently implicated in leukemogenesis [20]. However, miRNA profiling of the two DLBCL lines U2932 and SUDHL5 indicated high levels of miR-142-3p and-5p, both in the total miRNA count as well as a strong increase of both mir-142-3p and -5p in the miRNA processing Ago-2 complex [34], that harbors the biologically active miRNAs of a given cell [35]. Likewise, high levels of miR-142 were also found in various leukemias [29].

MiR-142 is highly expressed in hematopoietic cells [36,37] and is also one of the miRNAs with the highest relative proportion within the Ago-complex in DLBCL cell lines [34]. Because miR-142 is the only human microRNA gene where mutations have consistently been found in tumors, we wanted to determine the impact of miR-142 inactivation on protein expression of DLBCL cell lines. To this end, we functionally deleted miR-142 by CRISPR/Cas9 knockout [38] in the two GC-DLBCL lines BJAB and SUDHL4 [39].

## 2. Materials and Methods

### 2.1. Cells

BJAB cells were cultured in RPMI media supplemented with 10% FCS and 0.5% antibiotics. SUDHL4 cells were also cultivated in RPMI 1640 medium (Sigma, Munich, Germany) supplemented with 10% (*v*/*v*) fetal calf serum (FCS; Biochrom, Berlin, Germany) and antibiotics (40 IU/mL penicillin and 50µg/mL Streptomycin [Sigma, Munich, Germany], 1 IU/mL Neomycin-sulphate [Roth, Karlsruhe, Germany], and 90 IU/mL Nystatin [Fagroms, Barsbüttel, Germany]). Human HEK 293T cells were purchased from the German collection of microorganisms and cell cultures (DSMZ) and authenticated using STR DNA typing. HEK 293T cells were cultured as described previously [40].

### 2.2. Generation of Knockout Cells Using CRISPR/Cas 9

For generation of the miR-142 knockout cells, guide RNA (gRNA) sequences were designed using the tools https://chopchop.cbu.uib.no/ (accessed in October 2015). The plasmids with the designed gRNAs were synthesized by Genescript (https://www.genescript.com (accessed in October 2015) in the backbone of pSPCas9(BB)-2A-GFP (PX458). The following 3 gRNAs were used in this work to generate a knockout of miR-142: gRNA1 5′ GAAAGCACTACTAACAGCAC 3′, gRNA 2 5′ AGTACACTCATCCATAAAGT 3′ and gRNA 3 5′ AGTAGTGCTTTCTACTTTAT 3′. The position of the mutations within the seed sequence of miR-142-3p and -5p (mutants 142-3p-M1 and miR-142-5p-M3) as well as the exact location of the gRNAs in respect to the miR-142 gene are shown in Supplementary Figures S1 and S2, respectively. Four days after electroporation, single green fluorescent cells were sorted into single wells of 96-well plates cells using a MoFlo-XDP cell sorter (Beckman Coulter, Krefeld, Germany) [41]. Isolated outgrowing clones were screened by sequencing of genomic PCR-amplicons to confirm the knockout. Cells that showed miR-142 deletion were additionally analyzed by Northern blotting.

### 2.3. RNA Isolation and Northern Blotting

Total cellular RNA was isolated from the cells using the miRNeasy kit (Qiagen, Hilden, Germany) according to the manufacturer’s instructions. RNA concentration and integrity were analyzed using a NanoDrop ND 2000 instrument (Thermo Scientific, Dreieich, Germany) and an Agilent 2100 instrument (Agilent small RNA kit, catalogue no. 5067-1548, Beutelsbach, Germany) as described before [42]. Northern blotting was carried out as described before [34]. Briefly, 5–10 µg total RNA was separated on a 12% Urea Acrylamide Gel (National Diagnostics, Beutelsbach, Germany). RNA was then transferred to a nylon membrane and chemically cross-linked using EDC. For the detection of miRNA-specific RNA, complementary oligomer probes for miR-142-3p: GAGACAGGTCCATAAAGTAGGAAACACTACA and -5p: GAGACAGGAGTAGTGCTTTCTACTTTATG [16] were radiolabelled using the mirVana kit (Life technologies). The underlined sequences represent the T7 anchor sequence. Hybridization was carried out at 55 °C overnight. After washing, blots were exposed to a storage Phosphorscreen for 24 h and visualized using a PhosphoImager (GE Healthcare Life Sciences, Freiburg, Germany). Blots were also analyzed for the expression of miR-21 and U6 RNA as a loading control using appropriate probes.

### 2.4. Growth and Cell Cycle Analysis of Knockout vs. Wild-Type Cells

10.000 cells/mL of the wild-type and 3 clones of the knockout cells were seeded in 96-well plates on day 1 and then counted on day 6, 9 and 14. The cell cycle state of 3 knockout clones vs. the wild-type cells was determined by propidium iodide staining. Propidium iodide solution (Product number 556463) was obtained from BD Sciences (Heidelberg, Germany) and used according to the manufacturer’s instructions. Cell cycle analysis was performed on a BD FACSLyric flow-cytometer using BD FACSuite software v.1.4.0.7047, followed by data analysis using FlowJo software 10.6.2.

### 2.5. Proteome Analysis

Cell pellets (5 biological replicates per cell type) were lysed in 8 M Urea/0.4 M NH_4_HCO_3_ by 5 min sonication (Sonopuls GM3200 with BR30 cup booster, Bandelin, Berlin, Germany). For further lysis, samples were centrifuged through QIAshredder homogenizers (QIAGEN GmbH, Hilden, Germany). Protein concentration was determined using a Bradford assay [43]. To ensure a maximum of reproducibility, all lysates were adjusted to a protein concentration of 2 µg/µL using 8 M Urea/0.4 M NH_4_HCO_3_. A total of 10 µg of protein was reduced using dithioerythritol (final concentration 5 mM) for 30 min at 37 °C. Subsequent alkylation of cysteines was performed with iodoacetamide (final concentration 15 mM) for 30 min in the dark. Prior to tryptic digestion, samples were diluted with H_2_O to give 1 M Urea. Digestion was performed using 200 ng of modified porcine trypsin (Promega, Madison, WI, USA) at 37 °C. A total of 1 µg of each sample was injected in an Ultimate 3000 nano-chromatography system (ThermoFisher Scientific, Waltham, MA, USA) and transferred to a trap column (Acclaim PepMap 100 µm × 2 cm, 5 µm particles, 100 Å, ThermoFisher Scientific). Separation was performed at 250 nL/min using a 50 cm reversed-phase separation column (PepMap RSLC C18 2 µm 100 Å 75 µm × 50 cm, Thermo Fisher Scientific). Solvent A was 0.1% formic acid in water and B 0.1% formic acid in acetonitrile. The chromatography method consisted of a 160 min gradient from 3% to 25% and a second 10 min gradient from 25% to 40% solvent B. Eluting peptides were analyzed on a Q Exactive HF X mass spectrometer (Thermo Fisher Scientific) with a top 15 data-dependent method. Data processing, peptide search and quantification were conducted with MaxQuant (v.1.6.7.0) [44] and the human subset of the UniProt database. Results were filtered to give a false discovery rate of <1%. Statistics, principal component analysis as well as hierarchical cluster, volcano plot, and scatter plot analyses were performed in Perseus (v.1.5.3.2) [45]. Bioinformatical analysis was carried out using two web-based tools DAVID [46,47] and STRING [48], the R statistical software (v.4.0.4; R Core Team 2021), and also via manual annotating.

### 2.6. mRNA-Microarray

Gene expression profiles of the wild-type and mutant cell lines were measured using SurePrint G3 Human Gene Expression 8 × 60 Kv2 Microarray (Agilent Technologies, Santa Clara, CA, USA) following the manufacturer’s protocol and are described in detail elsewhere ([49] and references therein). Background-corrected expression values were extracted using Agilent Feature Extraction Software. Data was log-transformed and quantile normalized using Agilent GeneSpring Software. Fold-changes for all genes were calculated as ratio of mutant vs. wild-type.

### 2.7. DNA Sequencing and Analysis

Whole genome sequencing DNA of the knockout vs. wild-type cells was performed using MGIEasy DNA Library Prep Kit and BGISEQ-500RS High-throughput Sequencing Set PE100 with 1 µg DNA input according to the manufacturer’s instructions. In short, genomic DNA of wild-type and knockout cells was fragmented to 350 bp using a Covaris Focus-Ultrasonicator and purified using magnetic beads. After end repair and tailing, adapters were ligated to the 5′ and 3′ end of the DNA using barcoded adaptors. DNA fragments were PCR amplified for 6 cycles and purified using magnetic beads. Equimolar pools of PCR fragments of the wild-type and knockout samples were generated and circularized. Subsequently, DNA nanoballs (DNBs) were generated using rolling circle amplification, loaded onto the flow cell and sequenced on a BGISEQ-500RS sequencer, generating paired end 100 bp reads.

The sequences were then compared to detect changes such as the deletion of the miR-142 coding sequence and also analyzed with respect to possible additional mutations that were incurred due to the CRISPR/Cas9 procedure. As mutations observed in the knockout lines appeared to affect transcription factor binding sites, these were analyzed using the publicly available FIMO software (https://meme-suite.org/meme/doc/fimo/html (accessed on July 2022). The mutant positions were analyzed with a window of +/−25 bp for transcription factor binding sites applying binding motives obtained from HOCOMOCO (https://hocomoco11.autosome.org/ (accessed on July 2022)). 

### 2.8. Expression and Reporter Vectors

The pSG5-miR-142-wt, -miR-142-M1 and -mir-142-M3 expression plasmids were described previously [16]. pSG5-miR-142-M1 features a point mutation in the seed sequence of miR-142-3p (5′-UGUAGUGU > CUUCCUACUUUAUGGA-3′) while -M3 has a point mutation in the seed sequence of miR-142-5p (5′-CAUAAAG > UUAGAAAGCACUACU-3′), as shown in Supplementary Figure S1. The 3′UTRs of AKT1S1, CCNB1, LIMA1 and TFRC were PCR-amplified using specific primers and ligated via SpeI and SacI restriction sites into the pMIR-RNL-TK vector described elsewhere [50]. The sequences of all specific cloning primers and the sequences of the amplified inserts, their genomic locations and NM identifiers of the target sequences are given in Supplementary Table S1.

### 2.9. Dual Luciferase Reporter Assays

The dual luciferase assays were performed as described recently [51]. In brief, 2–2.5 × 10^4^ HEK293T cells were seeded out per well of 96-well plate (Eppendorf, Hamburg, Germany) and transfected after 24 h with the respective combinations of reporter (50 ng/well) and expression vectors (200 ng/well) by the liquid handling system epMotion 5075 (Eppendorf, Hamburg, Germany). PolyFect transfection reagent (Qiagen, Hilden, Germany) was used for the transient transfections (48 h) and the Dual-Luciferase^®^ Reporter Assay System Kit (Promega, Mannheim, Germany) for conducting the dual luciferase assays. All dual luciferase assays were conducted in duplicates and were repeated 4 times.

### 2.10. Western Blotting

Western blotting was carried out exactly as described previously [40,52]. In short, cells were lysed in 2× sample buffer (130 mM Tris/HCl, 6% [*v*/*v*] SDS, 10% [*v*/*v*] 3-Mercapto-1,2-propandiol, 10% [*v*/*v*] glycerol) and 3 times sonicated for 3 s. A total of 30 µg/lane of cell extract was separated by gel electrophoresis and transferred to a nitrocellulose membrane. After blocking with 5% skim milk in PBS for 60 min at RT, the membranes were incubated at 4 °C overnight with primary antibody at a dilution of 1:1000 (rabbit anti-PKN2), 1:30 (rat anti-EZR) or 1:3000 (mouse anti-β–actin) in PBS–milk. After washing, the membranes were incubated for 60 min at 4 °C with appropriate secondary antibody coupled to horseradish peroxidase diluted 1:5000 in PBS–milk. Bound antibody was visualized using ECL [16]. PKN2 antibody (Cat. Nr. 8697) was purchased from Cell Signaling (Frankfurt/M., Germany); a rat monoclonal antibody (rat IgG2a) directed against Ezrin was described elsewhere (T. Pfitzner, MD thesis, Saarland University Medical School, 66421 Homburg/Saar, Germany, 1996). Mouse monoclonal antibody AC-15 against β-actin (Cat.Nr.: A5541; Merck-Sigma-Aldrich, Taufkirchen, Germany) served as loading control.

## 3. Results

### 3.1. Inactivation of miR-142 in the GC-DLBCL Lines SUDHL4 and BJAB by CRISPR/Cas9 Knockout

**Figure 1:**
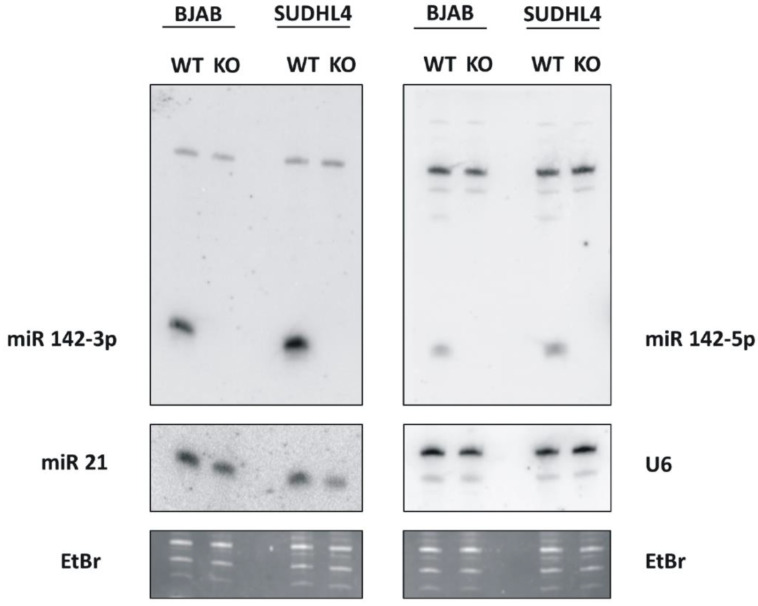
Analysis of miRNA expression in the knockout cells by Northern blotting. Total RNA (10µg/lane) from the BJAB-wild-type and SUDHL4-wild-type (“WT”) cells and the corresponding miR-142 knockout (“KO”) cells was separated on a 12% polyacrylamide gel, transferred to a nitrocellulose membrane and hybridized with ^32^P-labelled probe for miR-142-3p (left upper panel) or miR-142-5p (right upper panel). Bound probe was detected using a PhosphoImager. The bound probes were removed and the membranes were re-hybridized either with a probe to detect miR-21 (left side of middle panel) or U6 RNA (right side of middle panel) as indicated. The bottom panel shows the Ethidiumbromide (“Etbr”)-stained gels as loading control.

The aim of these experiments was to determine the contribution of miR-142 in the generation of DLBCL. MiR-142 was functionally eliminated in the GCB-DLBCL lines BJAB and SUDHL4. The guide RNAs used for CRISPR/Cas9 knockout [38] are shown in Supplementary Figure S2. Three days post-transfection, single clones were expanded, mutations were characterized by sequencing both strands with primers flanking the targeting region and analyzed by Northern blotting for the absence of detectable amounts of both miR-142-3p and -5p when grown in bulk culture. This data was also confirmed by whole genome sequencing. As depicted in Figure 1, both the BJAB and the SUDHL4 knockout clones showed no detectable miR-142-3p and -5p Northern blot signals as compared to the parental cell lines. As a control, the blots were stripped and re-analyzed for the expression of miR-21 as well as the U6 RNA. The wild-type and knockout cells both clearly exhibited signals for miR-21 and equal signals for the U6 RNA loading control.

### 3.2. Growth Properties of miR-142 Knockout Cells

**Figure 2:**
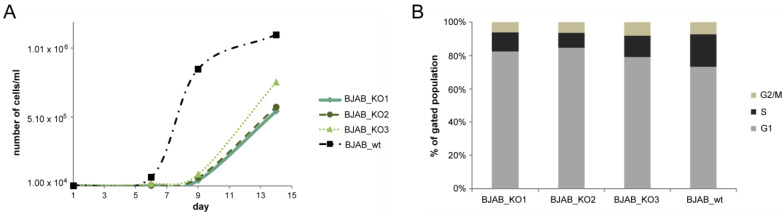
Growth properties of BJAB-miR-142 knockout clones. The BJAB wild-type (“wt”) and three knockout clones were seeded at 10.000 cells/mL and counted at the indicated days post-seeding (**A**). A cell cycle analysis of the BJAB wild-type cells and three knockout clones is shown in panel (**B**). The cells were stained with propidium iodide and analysed with a BD FACSLyric flow-cytometer.

The growth properties of three BJAB knockout or SUDHL4 knockout cell clones were analyzed in comparison to BJAB or SUDHL4 wild-type. The BJAB miR-142 knockout clones designated BJAB k.o.1, k.o.2 and k.o.3 were found to grow slower than the wild-type cells (Figure 2A), while there was no significant change in the growth of the SUDHL4 knockout vs. wild-type cells. A cell cycle analysis of three different BJAB knockout clones showed that a larger proportion of the wild-type BJAB cells were in S-phase as compared to the knockout-cells (Figure 2B, Supplementary Figure S3). As there was no significant change in the growth behavior of the SUDHL4 knockout vs. wild-type cells, these were not analyzed for their cell cycle status. The report by Mildner et al. (2017) showed that miR-142 deletion in knockout mice resulted in reduced growth of thymocytes with a concomitant increase in Cdkn1b (p27kip), and that the Cdkn1b 3′UTR is targeted by miR-142-3p [37]. We also found that the human CDKN1B 3′UTR is a target for miR-142-3p and that the responsibility of a 3′UTR reporter vector is lost when the 3′seed-sequence mutant M1 (shown in Supplementary Figure S1) is used. 

### 3.3. Analysis of Proteomic Alterations in BJAB and SUDHL4 Cell with Knockout vs. Wild-Type Genotype

A comparative proteome analysis of two lymphoma B-cell lines, SUDHL4 and BJAB, with either a miR-142 knockout or a wild-type genotype, was performed. Proteomes from five biological replicates per genotype per cell line were analyzed by nano LC-MS/MS.

**Figure 3:**
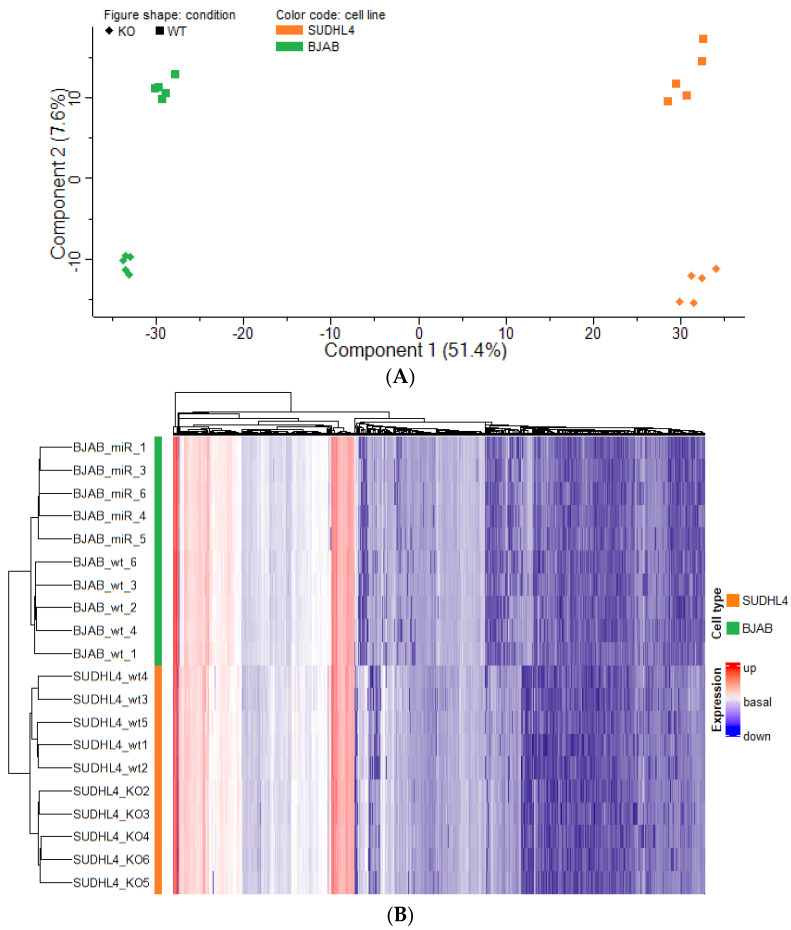
(**A**) Principal component analysis (PCA) of the proteome profiles from BJAB and SUDHL4 cell lines with either a wild-type or a miR-142 knockout genotype. Each data point represents a single biological replicate. Colors indicate the cell line, shapes for the genotype. (**B**) Heat map and unsupervised hierarchical clustering of protein intensity values from each of the replicate per cell line, and per genotype.

In total, 4863 proteins and 52,380 peptides were identified in the proteomes (Supplementary Table S2). The principal component analysis (PCA) showed separation according to cell type and genotype (Figure 3A), indicating major differences between the proteomes of the four groups. The heat map dendrogram shows cell line-wise grouping, regardless the genotype (Figure 3B). Within each of these sub-clusters, genotype-wise separation is distinctly visible.

### 3.4. Analysis of Proteomic Alterations in BJAB and SUDHL4 Cells with Knockout vs. Wild-Type Genotype

**Figure 4:**
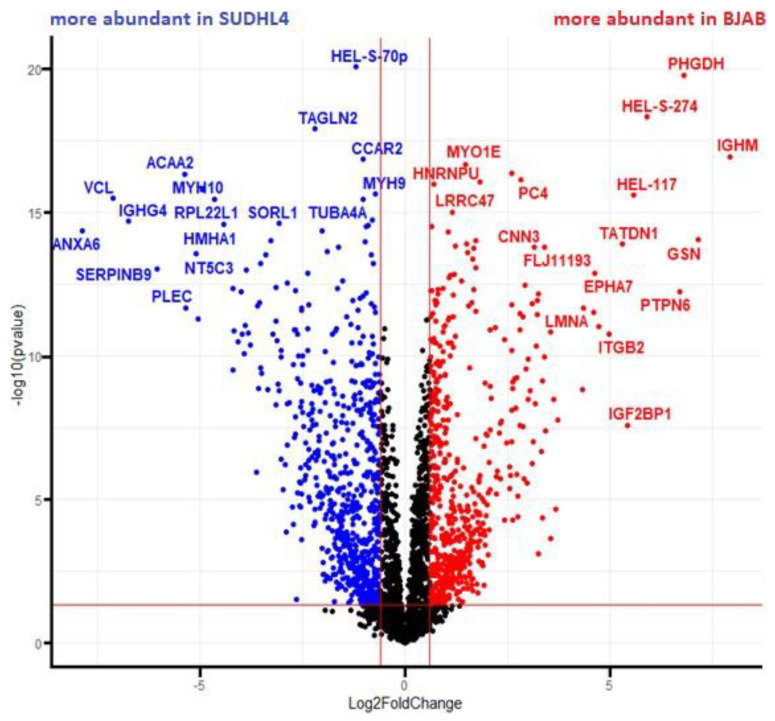
Volcano plot analysis of log2 normalized protein intensity values for BJAB/SUDHL4 cells. Non-paired t-test with false discovery rate (FDR) correction (0.05) was implemented. Each colored dot represents a protein fulfilling the significance criteria (|log2 FC| > 0.6; *p*-value < 0.05). For selected significant hits gene names are displayed.

First, a volcano plot analysis was performed, to elucidate general proteomic differences between both cell lines (Figure 4). This rendered 949 proteins more abundant in the SUDHL4 cell line (i.e., less abundant in BJAB) and 988 proteins more abundant in the BJAB cell line (i.e., less abundant in SUDHL4), regardless of the genotype. As expected, the result clearly demonstrates major differences between the proteome profiles of the two cell lines. 

**Figure 5:**
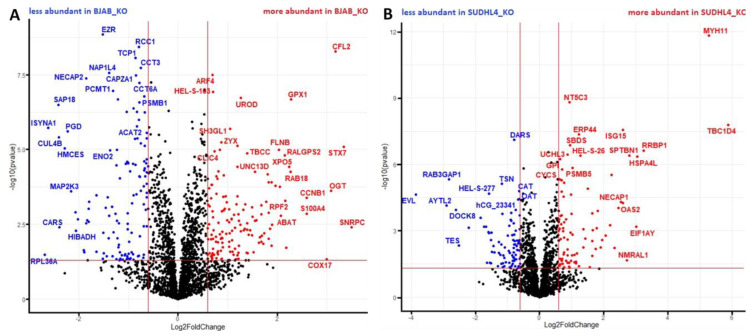
Volcano plot analysis of log2 protein intensity values for BJAB_KO/BJAB_WT (**A**) and SUDHL4_KO/SUDHL4_WT (**B**). Non-paired t-test with false discovery rate (FDR) correction (0.05) was implemented. Each colored dot represents a protein fulfilling the significance criteria (|log2 FC| > 0.6; *p*-value < 0.05). For selected significant hits gene names are displayed.

To analyze the effect of miR-142 knockout on both cell lines, additional volcano plot analyses were performed, for both cell lines separately (Figure 5A and Figure 5B, respectively). In BJAB, a total of 264 differentially abundant proteins were detected, out of which 123 were less abundant and 141 were more abundant in the miR-142 knockout genotype (BJAB_KO). The proteome of SUDHL4 showed a total of 170 significantly differentially abundant entities, in which 87 proteins were decreased and 83 proteins were increased in abundance in the miR-142 knockout genotype (SUDHL4_KO). Cell line specific differences of miR-142 knockout-induced proteome alterations became obvious.

### 3.5. Analysis of More and Less Abundant Proteins in Both Knockout Cell Lines

**Figure 6:**
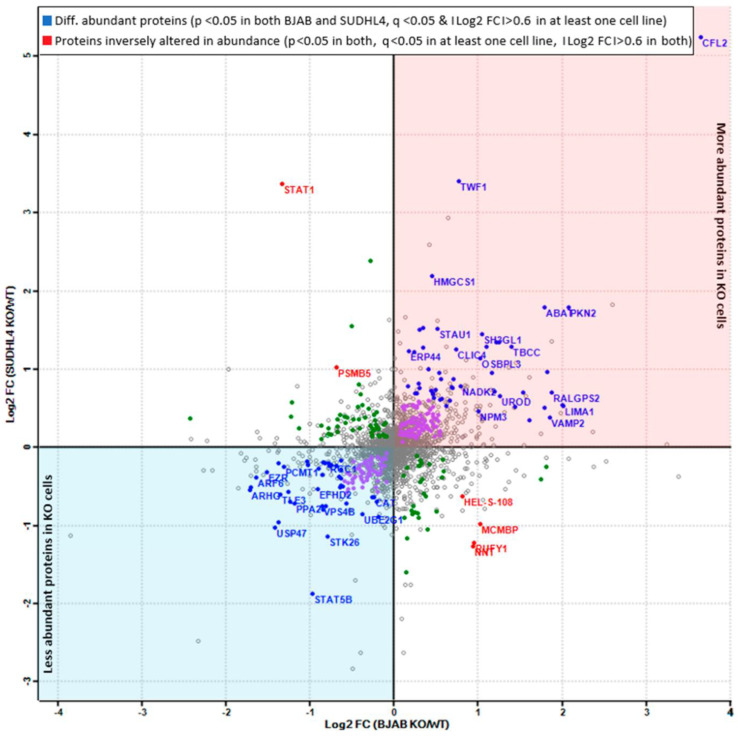
The scatter plot analysis displays the overlaps in more and less abundant protein groups in both cell lines. Filtering and overlap detection were performed upon a non-paired Student’s T-test, based on the following criteria: *p*-value < 0.05 for the same entry in both cell lines, q-value (i.e., FDR corrected *p*-value) <0.05 for the same entry in at least one of both cell lines, and |Log2FC| > 0.6 in both cell lines.

Given the prominent differences between BJAB and SUDHL4 cell lines, we looked for proteins which were altered in abundance in both miR-142 knockout cell lines. Therefore, a scatter plot analysis of log2 fold changes of differentially abundant proteins was performed (Figure 6) leading to the detection of 52 more and 41 less abundant proteins in both miR-142-knockout cell lines (Supplementary Table S3). The table further highlights the known and the novel miR-142 targets identified in this study and encompasses literature references indicating a role in promotion or suppression (oncogenic, suppressive, o/s) of cell growth. Of further interest, proteins with inversely altered abundance in BJAB vs. SUDHL4 were detected: STAT1, PSMB5, CCT6A, HEL-S-108, MCMBP, RUFY1, and NNT.

**Figure 7:**
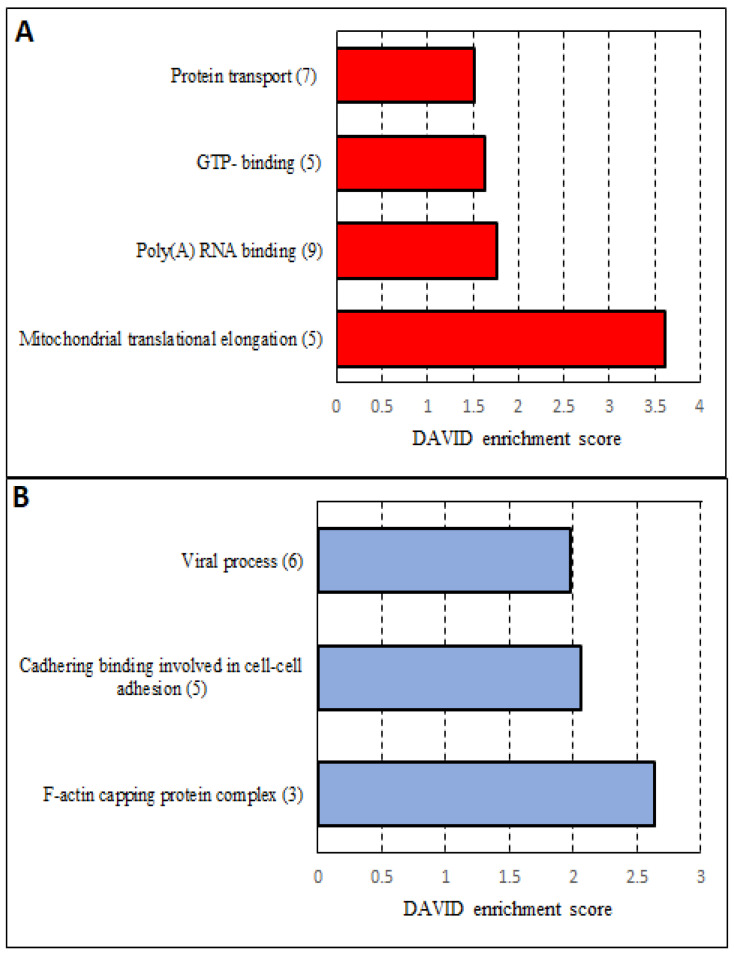
Overview of the results from the DAVID (Database for Annotation, Visualization, and Integrated Discovery) analysis of proteins which are more abundant (**A**) and less abundant (**B**) in knockout genotypes of both cell lines. The number behind each term indicates the number of proteins annotated to the named cluster. Results with an enrichment score >1.3 and the Benjamini value <0.01 were considered significant and were visualized. Categories used for the analysis were: GO biological process, GO molecular function, GO cellular component, UniProt keywords, Reactome Pathways and KEGG Pathways.

To gain insights into functions of the 93 proteins which were comparably altered in both cell lines, a DAVID functional annotation cluster analysis was carried out. It resulted in four significant clusters for the more abundant (Figure 7A) and in three for the less abundant proteins (Figure 7B). In the set of less abundant proteins in miR-142 knockout, proteins involved in cell–cell adhesion (PCMT1, CAPZB, CAPZA1, EFHD2, EZR), actin filament binding proteins (CAPZB, CAPZA1, CAPZA2) and of the F-actin-capping protein complex (CAPZB, CAPZA1, and CAPZA2) were found to be overrepresented. The involvement of miR-142 in actin filament homeostasis in megakaryocyte maturation had already been described in the miR-142 mouse knockout model mentioned above [36]. Proteins related to translational processes, including poly(A) RNA binding (YTHDF3, CSRP1, NUFIP2, HDLBP, SBDS, MRPL39, NPM3, MRPS5, MRPL44) and mitochondrial translational elongation (MRPS25, MRPL38, MRPL39, MRPS5, MRPL44), were found to be overrepresented in the group of proteins more abundant in the miR-142 knockout. Additionally, regulators of G proteins (GMPPB, ARF4, ARL3, RAB18, and TUBB4A) and transport proteins (STX12, ARF4, SEC23A, IPO9, ARL3, RAB18, and NAPG) appeared enriched in the set of up-regulated proteins.

**Figure 8:**
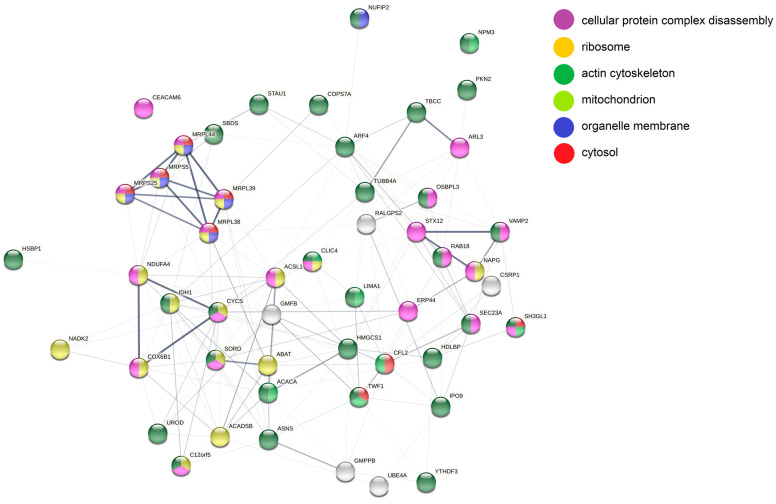
STRING analysis of proteins more abundant in both knockout cell lines. Nodes represent individual proteins and are colored based on the annotations placed at the right side of the figure. Line strength specifies confidence level from lowest (0.150) to highest (0.900).

Furthermore, the 93 differentially abundant proteins were analyzed with STRING (Figure 8). In line with the DAVID analysis, in the set of proteins more abundant in miR-142 cells a group of mitochondrial ribosomal proteins were interconnected by higher order (thicker) confidence nodes: MRPL44, MRPS5, MRPL39, MRPS25, and MRPL38. Furthermore, mitochondrial proteins of the respiratory chain, namely, the cytochrome c oxidase subunits NDUFA4 and COX6B1, as well as cytochrome c (CYCS), were forming a distinct cluster. Additionally, via manual annotation, the following proteins more generally related to “mitochondrion” were identified: NADK2, HEL-S-26/IDH1, HEL-S-95n/SORD, ABAT, ACADSB, TIGAR/C12orf5, ACSL1, and NAPG. Another prominent network of proteins related to “vesicular transport/trafficking” was identified and included STX12, VAMP2 and NAPG. Furthermore, COPS7A, a member of the COP9 signalosome [53] as well as NADK2, which also has oncogenic potential [54], were more abundant in both miRNA-142 knockout cell lines. 

**Figure 9:**
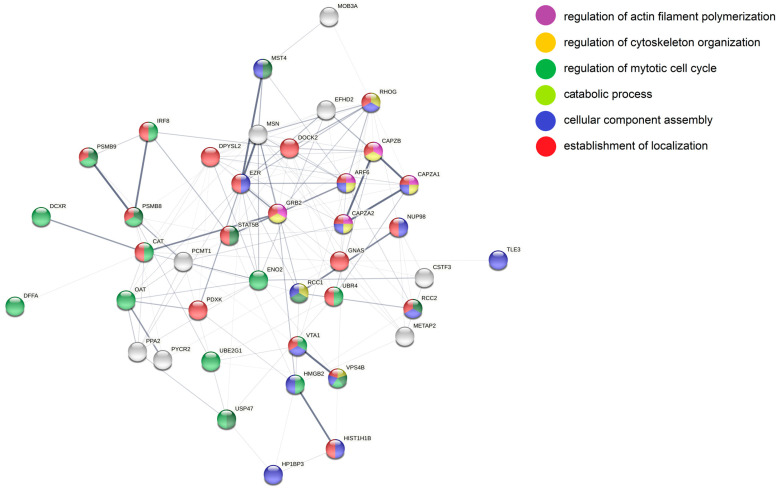
STRING analysis of proteins less abundant in both knockout cell lines. Nodes represent individual proteins and are colored based on the annotations placed at the right side of the figure. Line strength specifies confidence level from lowest (0.150) to highest (0.900).

In addition, for the less abundant proteins (Figure 9), the STRING analysis, similar to the DAVID analysis, resulted in a network of proteins related to the more general term “cytoskeleton/cytoskeletal reorganization”: MSN/HEL70, UBR4, EZR, RHOG, CAPZB, DPYSL2, DOCK2, and ARF6. Furthermore, clusters of proteins belonging to the family of F-actin-capping proteins (CAPZB, CAPZA1, and CAPZA2) and those manually annotated to “immunoregulation/immunoproteasome activity modulation” (PSMB8, PSMB9 and IRF8) were visible. Another series of proteins was grouped in a similar attempt according to its relation to “cell growth” and “cell growth-related signaling pathways”: GRB2, MST4/STK26, USP47, DPYSL2, and CAT. Finally, the proteins involved in “transcription/transcriptional activity regulation”, IRF8, NUP98, TLE3, HIST1H1B, STAT5B, HMGB2, USP47, and DOCK2 were displayed.

### 3.6. Analysis of mRNA Expression in miR-142 Knockout Cells

We then analyzed the gene expression profiles of the parental cell lines in comparison to one knockout clone each by microarray analysis [49]. The results are shown in Supplementary Table S4. Here, 19,924 genes were expressed in at least one of the analyzed samples. Of these, 235 displayed an at least a 2-fold increase and 388 genes showed an at least 1.5-fold increase in both miR-142 knockout lines. The results are shown in Supplementary Table S4. Of the genes that were at least 1.5-fold deregulated, 513 of those up-regulated in the BJAB knockout were down-regulated in the SUDHL4 cells while 496 of those that were up-regulated in the SUDHL4 knockout cells were down-regulated in the BJAB knockout cells. We then asked which of these genes de-regulated in the knockout cells were known or predicted target genes of miR-142 as determined using miRWalk2.0 (http://mirwalk.umm.uni-heidelberg.de/ (accessed on 26 October 2020) as shown in Supplementary Table S3. In this table, the already known targets and those established in the present analysis, are highlighted in yellow (column A). Table 1 summarizes the 20 most up- and down-regulated genes in the BJAB and SUDHL4 cell lines as well as the 20 most differentially expressed genes in the comparison of both miR-142 knockout lines.

We further determined which of the significantly up- or down-regulated proteins were also affected in the mRNA analysis. Here, we found that 35 of the 93 proteins were also changed in the microarray. Of these, 24 were up- and 11 were among the down-regulated genes/proteins. ERP44 (highlighted in blue; Supplementary Table S3, Column B), up-regulated in the proteome analysis, yielded no signal in the microarray. HMGB2 and DPYSL2, both reduced in abundance at the proteome analysis, were inversely regulated in that they were higher in the BJAB knockout but slightly reduced in the SUDHL4 knockout. These proteins are highlighted in blue and brown, respectively, in Supplementary Table S3 (Column B).

### 3.7. Target Genes of miR-142 Deregulated in DLBCL Knockout Cells

We questioned which of the 93 differentially abundant proteins in the two cell lines, identified by the proteome analysis, were known or predicted target genes of miR-142 as determined again using miRWalk2.0 (http://mirwalk.umm.uni-heidelberg.de/ (accessed on 26 october 2020) applying the following criteria: we consider a mRNA as a confirmed target when the responsiveness to miR-142 expression is lost in a luciferase assay by the mutation of respective miR-142 binding site or, when a miRNA-inhibitor of miR-142 (antisense-miR-142) inhibits miR-142 function [16]. The study by Chapnik et al. (2014) [36] identified Twinfilin (TWF1) as well as Cofilin (CFL2), and also BOD1 and ITGAV as novel targets for miR-142. These proteins were up-regulated by at least 1.5-fold in their analysis and we also found an at least 1.5–2-fold induction for these mRNAs in our analysis. The mRNA of the miR-142-targets WASL and GRIF2, up-regulated in the knockout model in that study [36], was not changed in our array. A study by Mildner et al. (2017) [37] had shown that mouse Cdkn1B is a target for miR-142 in T-cells; here, the human CDKN1B mRNA was present but not significantly changed (see below). From our array analysis, we chose as additional targets overlapping with the proteomic dataset CCNB1 (significantly increased in the proteome of BJAB knockout cells), LIMA1 and TFRC as these are potential targets of miR-142-5p, AKT1S1 (exclusively identified in both knockout cell lines) as a potential target of miR-142-3p. AKT1S1 (alternatively called PRAS40) was a very promising candidate because it is part of the mTORC1 complex which is a target for treatment of DLBCL [55]. 

**Figure 10:**
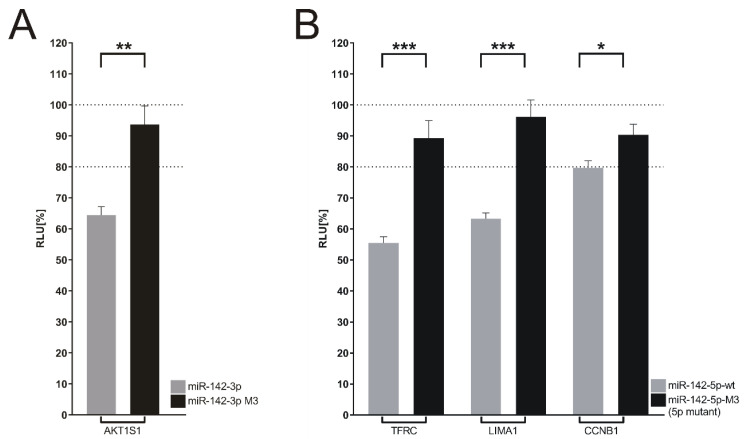
Confirmation of novel miR-142 targets by luciferase assay. (**A**) Analysis of AKT1S1 as a target of miR-142-3p. The 3′UTR of AKT1S1 was tested by dual-luciferase assay using either empty effector plasmid, effector plasmid expressing miR-142-wt or the miR-142-3p-M1 mutant which features a 3P-seed-sequences mutation (see Figure S1). The value obtained with the empty effector plasmid was set to 100%. (**B**). Analysis of TFRC, LIMA1 and CCNB1 as targets of miR-142-5p. The 3′UTRs of TFRC, LIMA1 and CCNB1 were assayed with empty effector plasmid, effector plasmid expressing miR-142-wt or the miR-142-3p-M3 mutant which features a 5P-seed-sequences mutation (see Figure S1). The value obtained with the empty effector plasmid was set to 100%. The data shown in (**A**,**B**) represent 4 independent assays carried out in duplicate. Three asterisks represent a significant reduction of the luciferase activity with a *p*-value ≤ 0.001. Two asterisks represent a significant reduction of the luciferase activity with a *p*-value ≤ 0.01 and ≥0.001. One asterisk represents a significant reduction of the luciferase activity with a *p*-value ≤ 0.05.

The potential miR-142-3p target AKT1S1 as well as the potential miR-142-5p targets CCNB1, LIMA1 and TFRC were analyzed by dual luciferase assay as described above. The RLUs (relative light units) of the four 3′UTR reporter constructs were clearly down-regulated with the miR-142-wt expression vector, while the corresponding -3p or -5p seed-sequence mutants failed to down-regulate their respective targets [16]. These results are shown in Figure 10A,B, respectively. Our approach identified novel targets of miR-142-3p as well as -5p and also shows that seed-sequence mutants can be used to validate miRNA binding sites within target mRNAs. This might be particularly useful when multiple potential targets of a given miRNA have to be confirmed. 

### 3.8. Western Blot Analysis of PKN2 and Ezrin

**Figure 11:**
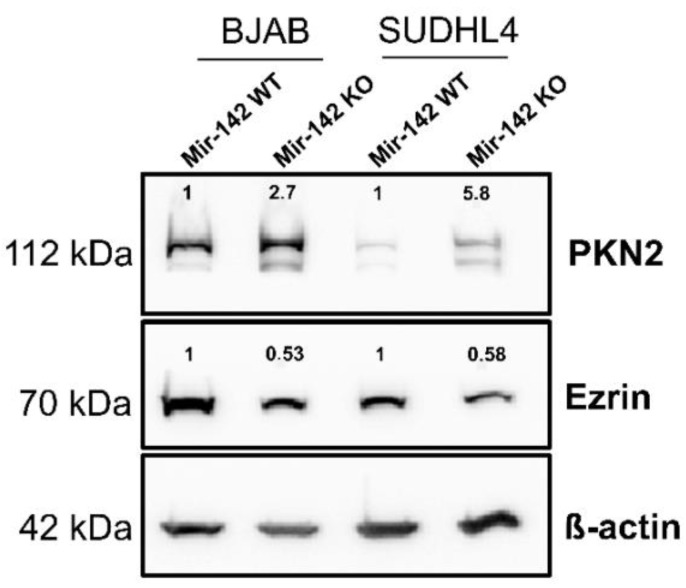
Western blot analysis: Extracts of BJAB-wild-type and SUDHL4-wild-type (“WT”) cells and the corresponding miR-142 knockout (“KO”) cells were separated on a 10% polyacrylamide gel, transferred to a nitrocellulose membrane and incubated with antibodies against PKN2, Ezrin and β-actin as a loading control. The membranes were incubated with the appropriate secondary antibodies coupled to horseradish peroxidase. Bound secondary antibodies were visualized by ECL. The bands were quantified using Image Lab 6.0.1. The mean value of two separate experiments is shown above the bands. The wild-type value was set to 1. The uncropped blots are shown in Figure S4.

The protein kinase PKN2, a potential tumor suppressor in colon carcinoma cell lines [56], was strongly up-regulated in the proteome analysis of both knockout cell lines, while Ezrin (EZR; Supplementary Table S3), a cytoskeleton protein with oncogenic potential [57], was moderately down-regulated. To corroborate these results, Western blots were carried out to compare the protein levels in the wild-type vs. knockout cells. As shown in Figure 11, PKN2 was strongly up-regulated in the knockout cells while Ezrin levels were reduced. These data confirmed the results of the proteome analysis for these two proteins. 

## 4. Discussion

The aim of this study was (i) to decipher actions of miR-142 at the proteome level, which may contribute to the induction or maintenance of DLBCL, (ii) to identify novel targets of miR-142-3p and -5p, and (iii) to show that seed-sequence mutants of miR-142-5p can be used to confirm a potential target of this miRNA. The successful generation of two miR-142 knockout GC-DLBCL lines was confirmed by DNA sequence analysis of the knockout cells and Northern blot analyses. As outlined above, miR-142 is considered to have tumor-suppressive functions in line with the loss-of-function mutations described for miR-142 mutants found in lymphoma and leukemia [20,58], in particular DLBCL [17,34]. For instance, reduced levels of miR-142-3p were found in acute myeloid leukemia [59]. However, elevated levels of this miRNA have been found in various lymphoma [17,19]. Further, the high abundance and thus functional relevance due to its relative increase in the Ago2-containing RISC complexes in DLBCL [34] are counterintuitive to its tumor-suppressive role. The contribution of this miRNA to the induction or maintenance of lymphoma remains thus unclear. Although the proteomics results also did not finally clarify the underlying mechanisms, the presented dataset gives interesting insight into miR-142 action in two different GC-DLBCL cell lines that were chosen for the knockout analysis because the miR-142 mutations described by Hezaveh et al. (2016) were found in GC-DLBCL [17]. For instance, various proteins known to have growth-inductive as well as -retarding properties are similarly altered in abundance in both miR-142 knockout cell lines, of which the majority of the reduced proteins are known to have growth-promoting potential (Supplementary Table S3). The reduced growth of the BJAB knockout cells, as compared to the wild-type was also counter-intuitive in respect to its growth-retarding role. It might in part be explained by the reduction of the proteins with growth-promoting properties in the knockout cells. Of further interest, proteins with inversely altered abundance in BJAB vs. SUDHL4 were detected. STAT1, PSMB5 and CCT6A were less abundant in the BJAB knockout cells than in the wild-type cells, while they showed increased abundance in the SUDHL4 knockout cells as opposed to the wild-type cells. Conversely, HEL-S-108/TPMS4, MCMBP, RUFY1 and NNT had higher abundance in the BJAB knockout cells but lower abundance in the SUDHL4 cells as opposed to the respective wild-type cells. The growth of highly aggressive ABC- as well as GC-DLBCL is induced by PD-L1 via JAK2-STAT1 (or STAT3) [60]. Likewise, high expression of CCT6A confers an unfavorable outcome in various tumor entities [61] such as breast [62] or cervical cancer [63], and CCT6A is secreted in extracellular vesicles [61]. The role of PSMB5 is less clear, as a series of 92 primary DLBCL showed no sign of this protein in the tumor cells, except within the microenvironment of the tumor [64], which could be explained with a possible secretion from the tumor cells into the stroma. 

Because miR-142 is among the most highly expressed miRNAs in DLBCL lines and is highly abundant in the Ago2-containing RISC complexes [34], it is thus not surprising that a relatively large number of proteins was deregulated upon its inactivation in the DLBCL lines. The fact that besides the up-regulated proteins, we found a relatively large number of proteins decreased in abundance indicates that miR-142 is part of a larger network of regulatory factors that conversely inhibit the expression of certain proteins/genes. For instance, the elimination of the highly expressed miR-142 in DLBCL [34] may result in its replacement in the Argo2- complexes by other endogenous miRNAs with additional impact on protein expression. 

In the microarray analysis, we found that 235 and 388 genes showed an either 2 or 1.5-fold increase or decrease, respectively, in the knock-out cells. A comparison of the overlap with the proteome analysis revealed that 24 of the mRNAs did increase in abundance of the 52 proteins (46.1%), while 11 mRNAs vs. 41 proteins (26.8%) also showed a decrease. The binding of a miRNA to a mRNA may result in a decrease of protein synthesis without affecting the mRNA stability while, alternatively, the binding to the mRNA may induce the degradation of the target [65]. 

Strikingly, in the proteomics screen we found a strong up-regulation of the actin filament remodeling factor Cofilin-2 (CFL2) and the actin-binding Twinfilin-1 (TWF1) protein (Supplementary Table S3). An important role for miR-142 in the actin filament homeostasis has been previously described in a mouse knockout model [36] where CFL2 and TWF1 were also found to be up-regulated. TWF1, CFL2 and Ezrin (EZR; the latter being moderately down-regulated in the knockout cells and CFL2 strongly up-regulated in the knockouts) belong to the large group of actin-binding proteins involved in the remodeling of actin filaments relevant for cell motility in cancer cells [66]. Additionally, the deregulated PSMB8 and -9 proteins also play a role in actin-filament reorganization. The down-regulated CAPZA1, CAPZB, EFHD2, EZR and PCMT1 proteins are involved in cell–cell adhesion; their reduced levels are compatible with the metastatic behavior of lymphoma cells. Furthermore, we found PKN2 to be more abundant in the miR-142 knockout cells in the proteome analysis and mRNA microarray. This observation was further confirmed by Western blot analysis in both knockout cell lines. The Ser/Thr kinase PKN2 is a potential target for both miR-142-3p and -5p (Supplementary Table S3). 

Various proteins associated with cytoskeleton remodeling were altered in abundance due to the knockout: Ezrin (EZR), as well as Cofilin (CFL2), are phosphorylated and activated by the Rho kinase ROCK1 [67] as well as their main targets, the Rho GTPases such as CDC25B which activate the cell cycle transition via PKN2 [68]. PKN2 is a potential target for miR-142-3p and -5p, which is in line with its strong induction in the knockout cell proteomes. Upon activation, PKN2 appears to play a stimulatory proliferative role in G2/M transition [68] in line with its pro-tumorigenic properties [69]. PKN2 phosphorylates and thereby activates mTORC1 to increase nutrient supply [70]. Subsequently, mTORC1 regulates mitochondrial functions as well as mRNA translation and ribosomal biogenesis [71]. MTORC1 in turn is complexed with AKT1S1, which we also identify as a novel target of mir-142-3p. A causal linkage of only one target gene like Ezrin or PKN2 to a complex cellular mechanism like proliferation is difficult. The impact of Ezrin or PKN2 on proliferation can probably be overcome by several other direct or indirect effects caused by dysregulation of multiple other target genes of miR-142. The down-regulated Histone H1.5 is tumor-suppressive in lymphoma and its loss in the germinal center B-cells induces activation of otherwise silent chromatin [72], but appears to be tumor-promoting in prostate carcinoma [73]. Cofilin (CFL2) showed the strongest up-regulation in both knockout lines and activation of the ROCK/LIMK/Cofilin pathway with a concomitant depletion of CAPZB and CAPZA2 results in increased CFL2 phosphorylation and enhanced cell invasion [74]. In line with this notion, the F-actin binding proteins CAPZB, CAPZA1 and CAPZA2 were down-regulated in the knockout cells. CAPZB has oncogenic potential [75], CAPZA1 inhibits cancer cell migration in gastric carcinoma [76] while CAPZA2 promotes gastric cancer cell migration and invasion [77]. 

The gene ontology analysis of the genes up-regulated in both knockout cell lines indicated that mitochondrial ribosomal proteins, among other factors found in mitochondria, were affected. For instance, the mitochondrial ribosomal proteins MRPL38, -39, and -44, were among the up-regulated proteins. Although their up-regulation, at first sight, appears not to be compatible with reduced growth, it was shown that some of these, such as MRPL17 (exclusively identified in both knockout cell types), -33, -35 or -44 (increased as a trend in both cell types, *p* < 0.05) were up-regulated in various tumors, such as glioma-, leukemia-, breast-, colon- or lung carcinoma (reviewed in [78]). COPS7A, a protein of the COP9 signalosome, was also induced. It is known that COP9 proteins, when expressed asynchronously from the other members of the COP9 signalosome, might contribute to cancer induction [79]. Lastly, COX6B1, NDUFA4 as well as cytochrome c (CYCS) of the mitochondrial respiratory chain were also induced, indicating a role for their regulation, directly or indirectly, by miR-142 with a possible link to the deregulation of the MRPL and MRPS proteins. The up-regulated mitochondrial NADK2 kinase is tumor-promoting [54] and is up-regulated in the knockout cells alongside with further proteins associated to mitochondrial function (HEL-S-26/IDH1, HEL-S-95n/SORD, ABAT, ACADSB, TIGAR/C12orf5, ACSL1, NAPG; see Figure 8 and accompanying text). Interestingly, mitochondrial-associated ER membranes under inflammatory stress are enriched for both miR-142-3p and -5p [80].

The PSMB8 and PSMB9 proteins, which are part of the 26S proteasome, were down-regulated. The 26S proteasome is a major constituent of the immune response which processes foreign proteins for presentation to immune cells by MHC-I [81]. Although their reduced levels in the two knockout cell lines appears to be counter-intuitive, this observation is compatible with the disturbed hematopoiesis and a loss of immune-response to soluble antigens in miR-142 null mice due to the impaired MHC-I presentation described previously [32,58]. 

In addition to already known targets (e.g., CFL2, TWF1, STAU1 and CLIC4) of miR-142, we used luciferase reporter assays to show that several candidates from the -omics screens were indeed targets of miR-142. As previously shown for the miR-142-3p seed-sequence mutants [16], the miR-142-5p-M3 seed mutant can also be used to confirm a 3′UTR as a target since corresponding reporter constructs lost their responsiveness due to the mutation of the miRNA 5p-seed sequence. Applying this strategy, we could confirm LIMA1 (Eplin), CCNB1 (Cyclin B1), AKT1S1 and TFRC as novel miR-142-3p or -5p targets. Similar to CFL2, LIMA1/Eplin inhibits actin filament depolarization. However, low levels of LIMA1 are associated with tumor growth [82], and LIMA1 binds to the known miR-142 target RAC1 [83] while low levels of Cyclin B1 (CCNB1) are associated with reduced cell growth in accordance with a tumor-suppressive role of miR-142. CCNB1 is directly up-regulated by FOXM1 over-expression in DLBCLs [84]. The dampened regulation of CCNB1 by the loss of miR-142-5p in DLBCLs can contribute to the malignant phenotype. TFRC plays a role in mitochondrial morphology [85]. As a down-stream target of c-MYC, it contributes to lymphomagenesis [86] and, in conjunction with cadherins like CAPZA1 or EZR, regulates ferroptosis [87]. The up-regulated ACADSB protein already mentioned above is also involved in ferroptosis and high levels reduce cell growth [88]. 

Various reports described the secretion of miR-142 in exosomes isolated from serum of transplant patients [89], human T-lymphocytes of type I diabetes patients [90] or Sjögren’s syndrome [91], macrophages [92], or CD4+T-cells that activate heart myofibroblasts [93]. Exosome-derived miR-142-5p expressed in cervical squamous cell carcinoma inhibits the CD8+ T-cell-mediated immune response via induction of indoleamine-2,3-diogxigenase (IDO) in tumor-associated lymphatic vessels, thereby exhausting the CD8+ cells [94]. So far, the exosomal release of miR-142-3p or-5p from B-cells or B-lymphoma has not been reported. It is conceivable, however, that miR-142-3p and/or -5p are secreted from DLBCL cells to generate a microenvironment favorable to the growth of the tumor or as a means to evade the immune surveillance of the host. Increased levels of exosomal miR-142-3p are observed during allograft rejection and lead to down-regulation of RAB11FIP2 in endothelial cells after up-take of the miR-142-3p contained in the vesicles released from activated T-cells [89].

## 5. Conclusions

Our observations might explain why the knockout of miR-142 led to a reduced growth of the BJAB cells contrary to the expectation that the loss of a potentially tumor-suppressive miRNA should induce cell growth. As shown for the miR-142 knockout mice [20], knockout cell lines may be used to identify novel targets of (human) miRNAs. Lastly, seed-sequence mutants of a miRNA may be employed to screen larger numbers of potential 3′UTR targets as the time-consuming generation of binding site mutants can be avoided. In summary, we found that a large number of genes/proteins considered to have oncogenic properties were induced in the knockout cells in line with a tumor suppressive function of miR-142, which additionally enforces the notion that miRNAs might affect indirect layers of transcriptional regulation. Overall, the deletion of a highly expressed miRNA reveals the complex contribution of miRNAs within cellular regulatory networks.

## Figures and Tables

**Table 1:** The 20 most up- (dark grey) and down-regulated (light grey) genes in miR-142 knockout BJAB and SUDHL4 cell lines as well as the 20 most differentially expressed genes in the comparison of miR-142 knockout SUDHL4 vs. BJAB cells.

BJAB		SUDHL4		SUDHL4 vs. BJAB	
Gene Symbol	FC KO vs. WT	Gene Symbol	FC KO vs. WT	Gene Symbol	FC Difference
CASP1	0.002	TUBB8	0.004	CAMK2N2	−90.240
HEBP2	0.002	GPATCH1	0.005	APCDD1	−43.254
CARD16	0.003	PSCA	0.007	DAB2IP	−41.563
DCN	0.004	TTN	0.011	MAP3K6	−34.677
THEMIS	0.006	LMX1B	0.011	BCL2L15	−33.949
LRFN3	0.007	NRIP1	0.012	FBXL7	−32.339
SLC38A4	0.009	SLC6A2	0.013	ART5	−27.928
CD274	0.009	ENTPD8	0.014	GPR171	−24.575
ADCYAP1	0.009	CD200R1	0.016	H6PD	−23.742
OR51S1	0.011	CCDC120	0.016	CD3G	−23.354
CBS	0.011	NAV2	0.017	MORN4	−21.185
FAM183A	0.012	PCDH15	0.017	CELSR1	−19.788
PSG5	0.012	CCDC144A	0.017	GPRASP2	−19.247
SMOC2	0.012	FOXR1	0.017	C12orf40	−18.125
TRAPPC3L	0.013	SMPDL3A	0.019	FOS	−15.809
XAF1	0.016	ST8SIA1	0.019	TGM7	−14.890
CLIP4	0.016	RXFP1	0.020	AS3MT	−14.816
CARD17	0.016	PLXNB3	0.020	PYGL	−13.197
NT5E	0.020	DNASE2B	0.020	GUCA1A	−12.201
RIC3	0.020	OIT3	0.021	C16orf62	−11.145
PDCD1LG2	20.835	FOXL1	55.254	BDNF	37.896
PLEKHH2	20.971	FBXW2	57.711	TMPRSS15	39.814
PPP4R4	22.315	MAGEB16	58.134	WISP1	40.863
IGF2BP2	22.854	KIF5A	58.160	SEMA5A	43.383
COL4A4	23.466	GPR85	66.176	GJB1	46.221
FOXL1	25.999	FOXC2	68.900	PLAU	46.481
CHRD	27.345	TDRD6	70.648	SULF2	47.733
NPHP1	28.583	GAL3ST2	71.346	CNPY1	50.162
FBXL7	29.758	PAGE4	73.636	RSG1	51.428
IRS1	35.501	OR2H1	75.353	IFI44L	58.035
OPLAH	47.738	KITLG	78.372	AKAP14	63.312
DHRS9	52.841	CACNA1B	83.562	CLIP4	64.945
HOXB6	64.707	SDC4	98.925	MAGEB16	65.662
AIM2	66.156	SKI	102.922	FOXC2	71.354
TCEAL8	67.141	LOC100505841	103.228	OR2H1	80.166
CAMK2N2	84.172	VAMP3	112.579	NT5E	81.031
RIMKLA	96.113	ZNF680	262.647	OR51S1	94.888
ACAP2	127.043	LRRC1	2774.524	XAF1	95.632
ARMCX5	144.324	ZNF521	2876.359	CACNA1B	105.876
TCEAL8	158.819	KCNC2	3110.916	SLC38A4	120.136

## Data Availability

The mass spectrometry proteomics data have been deposited to the ProteomeXchange Consortium via the PRIDE [95] partner repository with the dataset identifier PXD037148.

## Supplementary Materials

The following supporting information can be downloaded at: https://www.mdpi.com/article/10.3390/cancers14205031/s1, Table S1: Cloning primers; Table S2: Raw data of proteome analysis; Table S3: Comparison mRNA microarray vs. proteome analysis vs. miR-142 target prediction; Table S4: Raw data mRNA microarray. Figure S1: Point mutations M1 and M3 in miR-142. Shown is the secondary structure of pri-miR-142. Mutations M1 and M3 occurring in DLBCL tumors studied in this work are indicated in red and annotated as mut 1 and 3. Mut 1 (U>C) is found in the seed region of miR-142-3p. Mut 3 (G>U) is in the seed of miR-142-5p. The mature miRNA species are shown in blue and the seed sequences in dark blue. The secondary structure was generated by using the RNAfold web server of the University of Vienna: (http://rna.tbi.univie.ac.at/cgi-bin/RNAWebSuite/RNAfold.cgi); Figure S2: (A) Genomic sequence of pri-miR-142. The positions of the mature miR-142-3p and -5p are underlined. The positions of the gRNAs are shown as red arrows. (B) Sequences of the three gRNAs used; Figure S3: Cell cycle analysis of the three BJAB knockout cell lines vs. BJAB-wild type cells. Cell cycle analysis was carried out by FACS analysis. Cells were stained with propidium iodide. The relative amounts of cells in G1, S and G2/M are shown in Figure 2B. Figure S4: uncropped blots of Figure 11.

## References

[1] Hanahan D., Weinberg R.A. (2011). Hallmarks of cancer: The next generation. Cell.

[2] Romano G., Veneziano D., Acunzo M., Croce C.M. (2017). Small non-coding RNA and cancer. Carcinogenesis.

[3] Bartel D.P. (2009). MicroRNAs: Target recognition and regulatory functions. Cell.

[4] Dueck A., Meister G. (2014). Assembly and function of small RNA—Argonaute protein complexes. Biol. Chem..

[5] Calin G.A., Croce C.M. (2006). MicroRNA signatures in human cancers. Nat. Rev. Cancer.

[6] Acunzo M., Romano G., Wernicke D., Croce C.M. (2015). MicroRNA and cancer—A brief overview. Adv. Biol. Regul..

[7] Costinean S., Zanesi N., Pekarsky Y., Tili E., Volinia S., Heerema N., Croce C.M. (2006). Pre-B cell proliferation and lymphoblastic leukemia/high-grade lymphoma in E(mu)-miR155 transgenic mice. Proc. Natl. Acad. Sci. USA.

[8] Hermeking H. (2010). The miR-34 family in cancer and apoptosis. Cell Death Differ..

[9] Welch C., Chen Y., Stallings R.L. (2007). MicroRNA-34a functions as a potential tumor suppressor by inducing apoptosis in neuroblastoma cells. Oncogene.

[10] Hayes J., Peruzzi P.P., Lawler S. (2014). MicroRNAs in cancer: Biomarkers, functions and therapy. Trends Mol. Med..

[11] Umu S.U., Langseth H., Bucher-Johannessen C., Fromm B., Keller A., Meese E., Lauritzen M., Leithaug M., Lyle R., Rounge T.B. (2018). A comprehensive profile of circulating RNAs in human serum. RNA Biol..

[12] Backes C., Meese E., Keller A. (2016). Specific miRNA Disease Biomarkers in Blood, Serum and Plasma: Challenges and Prospects. Mol. Diagn. Ther..

[13] Anastasiadou E., Jacob L.S., Slack F.J. (2018). Non-coding RNA networks in cancer. Nat. Rev. Cancer.

[14] Campo E., Swerdlow S.H., Harris N.L., Pileri S., Stein H., Jaffe E.S. (2011). The 2008 WHO classification of lymphoid neoplasms and beyond: Evolving concepts and practical applications. Blood.

[15] Imig J., Motsch N., Zhu J.Y., Barth S., Okoniewski M., Reineke T., Tinguely M., Faggioni A., Trivedi P., Meister G. (2011). microRNA profiling in Epstein-Barr virus-associated B-cell lymphoma. Nucleic Acids Res..

[16] Kwanhian W., Lenze D., Alles J., Motsch N., Barth S., Doll C., Imig J., Hummel M., Tinguely M., Trivedi P. (2012). MicroRNA-142 is mutated in about 20% of diffuse large B-cell lymphoma. Cancer Med..

[17] Hezaveh K., Kloetgen A., Bernhart S.H., Mahapatra K.D., Lenze D., Richter J., Haake A., Bergmann A.K., Brors B., Burkhardt B. (2016). Alterations of miRNAs and miRNA-regulated mRNA expression in GC B cell lymphomas determined by integrative sequencing analysis. Haematologica.

[18] Bouska A., Zhang W., Gong Q., Iqbal J., Scuto A., Vose J., Ludvigsen M., Fu K., Weisenburger D.D., Greiner T.C. (2017). Combined copy number and mutation analysis identifies oncogenic pathways associated with transformation of follicular lymphoma. Leukemia.

[19] Morin R.D., Assouline S., Alcaide M., Mohajeri A., Johnston R.L., Chong L., Grewal J., Yu S., Fornika D., Bushell K. (2016). Genetic Landscapes of Relapsed and Refractory Diffuse Large B-Cell Lymphomas. Clin. Cancer Res..

[20] Trissal M.C., Wong T.N., Yao J.C., Ramaswamy R., Kuo I., Baty J., Sun Y., Jih G., Parikh N., Berrien-Elliott M.M. (2018). MIR142 Loss-of-Function Mutations Derepress ASH1L to Increase HOXA Gene Expression and Promote Leukemogenesis. Cancer Res..

[21] Galka-Marciniak P., Urbanek-Trzeciak M.O., Nawrocka P.M., Dutkiewicz A., Giefing M., Lewandowska M.A., Kozlowski P. (2019). Somatic Mutations in miRNA Genes in Lung Cancer-Potential Functional Consequences of Non-Coding Sequence Variants. Cancers.

[22] Gauwerky C.E., Huebner K., Isobe M., Nowell P.C., Croce C.M. (1989). Activation of MYC in a masked t(8;17) translocation results in an aggressive B-cell leukemia. Proc. Natl. Acad. Sci. USA.

[23] Robbiani D.F., Bunting S., Feldhahn N., Bothmer A., Camps J., Deroubaix S., McBride K.M., Klein I.A., Stone G., Eisenreich T.R. (2009). AID produces DNA double-strand breaks in non-Ig genes and mature B cell lymphomas with reciprocal chromosome translocations. Mol. Cell.

[24] Fu Y., Sun L.Q., Huang Y., Quan J., Hu X., Tang D., Kang R., Li N., Fan X.G. (2018). miR-142-3p Inhibits the Metastasis of Hepatocellular Carcinoma Cells by Regulating HMGB1 Gene Expression. Curr. Mol. Med..

[25] Bandres E., Cubedo E., Agirre X., Malumbres R., Zarate R., Ramirez N., Abajo A., Navarro A., Moreno I., Monzo M. (2006). Identification by Real-time PCR of 13 mature microRNAs differentially expressed in colorectal cancer and non-tumoral tissues. Mol. Cancer.

[26] Wang Z., Liu Z., Fang X., Yang H. (2017). MiR-142-5p Suppresses Tumorigenesis by Targeting PIK3CA in Non-Small Cell Lung Cancer. Cell Physiol. Biochem..

[27] Mansoori B., Mohammadi A., Ghasabi M., Shirjang S., Dehghan R., Montazeri V., Holmskov U., Kazemi T., Duijf P., Gjerstorff M. (2019). miR-142-3p as tumor suppressor miRNA in the regulation of tumorigenicity, invasion and migration of human breast cancer by targeting Bach-1 expression. J. Cell. Physiol..

[28] Zhang X., Yan Z., Zhang J., Gong L., Li W., Cui J., Liu Y., Gao Z., Li J., Shen L. (2011). Combination of hsa-miR-375 and hsa-miR-142-5p as a predictor for recurrence risk in gastric cancer patients following surgical resection. Ann. Oncol..

[29] Dahlhaus M., Roolf C., Ruck S., Lange S., Freund M., Junghanss C. (2013). Expression and prognostic significance of hsa-miR-142-3p in acute leukemias. Neoplasma.

[30] Tan Y.F., Chen Z.Y., Wang L., Wang M., Liu X.H. (2020). MiR-142-3p functions as an oncogene in prostate cancer by targeting FOXO1. J. Cancer.

[31] Yang L., Wang Z.F., Wu H., Wang W. (2018). miR-142-5p Improves Neural Differentiation and Proliferation of Adipose-Derived Stem Cells. Cell. Physiol. Biochem..

[32] Kramer N.J., Wang W.L., Reyes E.Y., Kumar B., Chen C.C., Ramakrishna C., Cantin E.M., Vonderfecht S.L., Taganov K.D., Chau N. (2015). Altered lymphopoiesis and immunodeficiency in miR-142 null mice. Blood.

[33] Shrestha A., Carraro G., El Agha E., Mukhametshina R., Chao C.M., Rizvanov A., Barreto G., Bellusci S. (2015). Generation and Validation of miR-142 Knock Out Mice. PLoS ONE.

[34] Ayoubian H., Ludwig N., Fehlmann T., Menegatti J., Groger L., Anastasiadou E., Trivedi P., Keller A., Meese E., Grasser F.A. (2019). Epstein-Barr Virus Infection of Cell Lines Derived from Diffuse Large B-Cell Lymphomas Alters MicroRNA Loading of the Ago2 Complex. J. Virol..

[35] Flores O., Kennedy E.M., Skalsky R.L., Cullen B.R. (2014). Differential RISC association of endogenous human microRNAs predicts their inhibitory potential. Nucleic Acids Res..

[36] Chapnik E., Rivkin N., Mildner A., Beck G., Pasvolsky R., Metzl-Raz E., Birger Y., Amir G., Tirosh I., Porat Z. (2014). miR-142 orchestrates a network of actin cytoskeleton regulators during megakaryopoiesis. Elife.

[37] Mildner A., Chapnik E., Varol D., Aychek T., Lampl N., Rivkin N., Bringmann A., Paul F., Boura-Halfon S., Hayoun Y.S. (2017). MicroRNA-142 controls thymocyte proliferation. Eur. J. Immunol..

[38] Charpentier E., Doudna J.A. (2013). Biotechnology: Rewriting a genome. Nature.

[39] Deeb S.J., D’Souza R.C., Cox J., Schmidt-Supprian M., Mann M. (2012). Super-SILAC allows classification of diffuse large B-cell lymphoma subtypes by their protein expression profiles. Mol. Cell. Proteom..

[40] Hart M., Rheinheimer S., Leidinger P., Backes C., Menegatti J., Fehlmann T., Grasser F., Keller A., Meese E. (2016). Identification of miR-34a-target interactions by a combined network based and experimental approach. Oncotarget.

[41] Fruhwald J., Camacho Londono J., Dembla S., Mannebach S., Lis A., Drews A., Wissenbach U., Oberwinkler J., Philipp S.E. (2012). Alternative splicing of a protein domain indispensable for function of transient receptor potential melastatin 3 (TRPM3) ion channels. J. Biol. Chem..

[42] Hart M., Wach S., Nolte E., Szczyrba J., Menon R., Taubert H., Hartmann A., Stoehr R., Wieland W., Grasser F.A. (2013). The proto-oncogene ERG is a target of microRNA miR-145 in prostate cancer. FEBS J..

[43] Bradford M.M. (1976). A rapid and sensitive method for the quantitation of microgram quantities of protein utilizing the principle of protein-dye binding. Anal. Biochem..

[44] Cox J., Mann M. (2008). MaxQuant enables high peptide identification rates, individualized p.p.b.-range mass accuracies and proteome-wide protein quantification. Nat. Biotechnol..

[45] Tyanova S., Temu T., Sinitcyn P., Carlson A., Hein M.Y., Geiger T., Mann M., Cox J. (2016). The Perseus computational platform for comprehensive analysis of (prote)omics data. Nat. Methods.

[46] Sherman B.T., Hao M., Qiu J., Jiao X., Baseler M.W., Lane H.C., Imamichi T., Chang W. (2022). DAVID: A web server for functional enrichment analysis and functional annotation of gene lists (2021 update). Nucleic Acids Res..

[47] Huang da W., Sherman B.T., Lempicki R.A. (2009). Systematic and integrative analysis of large gene lists using DAVID bioinformatics resources. Nat. Protoc..

[48] Szklarczyk D., Gable A.L., Lyon D., Junge A., Wyder S., Huerta-Cepas J., Simonovic M., Doncheva N.T., Morris J.H., Bork P. (2019). STRING v11: Protein-protein association networks with increased coverage, supporting functional discovery in genome-wide experimental datasets. Nucleic Acids Res..

[49] Diener C., Hart M., Kehl T., Rheinheimer S., Ludwig N., Krammes L., Pawusch S., Lenhof K., Tanzer T., Schub D. (2020). Quantitative and time-resolved miRNA pattern of early human T cell activation. Nucleic Acids Res..

[50] Beitzinger M., Meister G. (2011). Experimental identification of microRNA targets by immunoprecipitation of Argonaute protein complexes. Methods Mol. Biol..

[51] Alles J., Menegatti J., Motsch N., Hart M., Eichner N., Reinhardt R., Meister G., Grasser F.A. (2016). miRNA expression profiling of Epstein-Barr virus-associated NKTL cell lines by Illumina deep sequencing. FEBS Open Bio.

[52] Alles J., Hasler D., Kazmi S.M.A., Tesson M., Hamilton A., Schlegel L., Marx S., Eichner N., Reinhardt R., Meister G. (2015). Epstein-Barr Virus EBER Transcripts Affect miRNA-Mediated Regulation of Specific Targets and Are Processed to Small RNA Species. Non-Coding RNA.

[53] Schlierf A., Altmann E., Quancard J., Jefferson A.B., Assenberg R., Renatus M., Jones M., Hassiepen U., Schaefer M., Kiffe M. (2016). Targeted inhibition of the COP9 signalosome for treatment of cancer. Nat. Commun..

[54] Tedeschi P.M., Bansal N., Kerrigan J.E., Abali E.E., Scotto K.W., Bertino J.R. (2016). NAD+ Kinase as a Therapeutic Target in Cancer. Clin. Cancer Res..

[55] Ezell S.A., Wang S., Bihani T., Lai Z., Grosskurth S.E., Tepsuporn S., Davies B.R., Huszar D., Byth K.F. (2016). Differential regulation of mTOR signaling determines sensitivity to AKT inhibition in diffuse large B cell lymphoma. Oncotarget.

[56] Cheng Y., Zhu Y., Xu J., Yang M., Chen P., Xu W., Zhao J., Geng L., Gong S. (2018). PKN2 in colon cancer cells inhibits M2 phenotype polarization of tumor-associated macrophages via regulating DUSP6-Erk1/2 pathway. Mol. Cancer.

[57] Li N., Kong J., Lin Z., Yang Y., Jin T., Xu M., Sun J., Chen L. (2019). Ezrin promotes breast cancer progression by modulating AKT signals. Br. J. Cancer.

[58] Berrien-Elliott M.M., Sun Y., Neal C., Ireland A., Trissal M.C., Sullivan R.P., Wagner J.A., Leong J.W., Wong P., Mah-Som A.Y. (2019). MicroRNA-142 Is Critical for the Homeostasis and Function of Type 1 Innate Lymphoid Cells. Immunity.

[59] Ramsingh G., Koboldt D.C., Trissal M., Chiappinelli K.B., Wylie T., Koul S., Chang L.W., Nagarajan R., Fehniger T.A., Goodfellow P. (2010). Complete characterization of the microRNAome in a patient with acute myeloid leukemia. Blood.

[60] Pascual M., Mena-Varas M., Robles E.F., Garcia-Barchino M.J., Panizo C., Hervas-Stubbs S., Alignani D., Sagardoy A., Martinez-Ferrandis J.I., Bunting K.L. (2019). PD-1/PD-L1 immune checkpoint and p53 loss facilitate tumor progression in activated B-cell diffuse large B-cell lymphomas. Blood.

[61] Macario A.J.L., Conway de Macario E. (2021). Chaperonins in cancer: Expression, function, and migration in extracellular vesicles. Semin. Cancer Biol..

[62] Xu W.X., Song W., Jiang M.P., Yang S.J., Zhang J., Wang D.D., Tang J.H. (2021). Systematic Characterization of Expression Profiles and Prognostic Values of the Eight Subunits of the Chaperonin TRiC in Breast Cancer. Front. Genet..

[63] Ma J., Yang L., Feng H., Zheng L., Meng H., Li X. (2021). CCT6A may act as a potential biomarker reflecting tumor size, lymphatic metastasis, FIGO stage, and prognosis in cervical cancer patients. J. Clin. Lab. Anal..

[64] Delforoush M., Berglund M., Edqvist P.H., Sundstrom C., Gullbo J., Enblad G. (2017). Expression of possible targets for new proteasome inhibitors in diffuse large B-cell lymphoma. Eur. J. Haematol..

[65] Beitzinger M., Peters L., Zhu J.Y., Kremmer E., Meister G. (2007). Identification of human microRNA targets from isolated argonaute protein complexes. RNA Biol..

[66] Tanaka K., Takeda S., Mitsuoka K., Oda T., Kimura-Sakiyama C., Maeda Y., Narita A. (2018). Structural basis for cofilin binding and actin filament disassembly. Nat. Commun..

[67] Hebert M., Potin S., Sebbagh M., Bertoglio J., Breard J., Hamelin J. (2008). Rho-ROCK-dependent ezrin-radixin-moesin phosphorylation regulates Fas-mediated apoptosis in Jurkat cells. J. Immunol..

[68] Schmidt A., Durgan J., Magalhaes A., Hall A. (2007). Rho GTPases regulate PRK2/PKN2 to control entry into mitosis and exit from cytokinesis. EMBO J..

[69] Murray E.R., Menezes S., Henry J.C., Williams J.L., Alba-Castellon L., Baskaran P., Quetier I., Desai A., Marshall J.J.T., Rosewell I. (2022). Disruption of pancreatic stellate cell myofibroblast phenotype promotes pancreatic tumor invasion. Cell Rep..

[70] Wallroth A., Koch P.A., Marat A.L., Krause E., Haucke V. (2019). Protein kinase N controls a lysosomal lipid switch to facilitate nutrient signalling via mTORC1. Nat. Cell Biol..

[71] de la Cruz Lopez K.G., Toledo Guzman M.E., Sanchez E.O., Garcia Carranca A. (2019). mTORC1 as a Regulator of Mitochondrial Functions and a Therapeutic Target in Cancer. Front. Oncol..

[72] Yusufova N., Kloetgen A., Teater M., Osunsade A., Camarillo J.M., Chin C.R., Doane A.S., Venters B.J., Portillo-Ledesma S., Conway J. (2021). Histone H1 loss drives lymphoma by disrupting 3D chromatin architecture. Nature.

[73] Khachaturov V., Xiao G.Q., Kinoshita Y., Unger P.D., Burstein D.E. (2014). Histone H1.5, a novel prostatic cancer marker: An immunohistochemical study. Hum. Pathol..

[74] Ohishi T., Yoshida H., Katori M., Migita T., Muramatsu Y., Miyake M., Ishikawa Y., Saiura A., Iemura S.I., Natsume T. (2017). Tankyrase-Binding Protein TNKS1BP1 Regulates Actin Cytoskeleton Rearrangement and Cancer Cell Invasion. Cancer Res..

[75] Ye Z., Wang D., Lu Y., He Y., Yu J., Wei W., Chen C., Wang R., Zhang L., Zhang L. (2021). Vacuolin-1 inhibits endosomal trafficking and metastasis via CapZbeta. Oncogene.

[76] Lee Y.J., Jeong S.H., Hong S.C., Cho B.I., Ha W.S., Park S.T., Choi S.K., Jung E.J., Ju Y.T., Jeong C.Y. (2013). Prognostic value of CAPZA1 overexpression in gastric cancer. Int. J. Oncol..

[77] Kwon M.J., Kim R.N., Song K., Jeon S., Jeong H.M., Kim J.S., Han J., Hong S., Oh E., Choi J.S. (2017). Genes co-amplified with ERBB2 or MET as novel potential cancer-promoting genes in gastric cancer. Oncotarget.

[78] Huang G., Li H., Zhang H. (2020). Abnormal Expression of Mitochondrial Ribosomal Proteins and Their Encoding Genes with Cell Apoptosis and Diseases. Int. J. Mol. Sci..

[79] Richardson K.S., Zundel W. (2005). The emerging role of the COP9 signalosome in cancer. Mol. Cancer Res..

[80] Wang W.X., Prajapati P., Nelson P.T., Springer J.E. (2020). The Mitochondria-Associated ER Membranes Are Novel Subcellular Locations Enriched for Inflammatory-Responsive MicroRNAs. Mol. Neurobiol..

[81] Kasahara M., Flajnik M.F. (2019). Origin and evolution of the specialized forms of proteasomes involved in antigen presentation. Immunogenetics.

[82] Nie Z., Du M.Q., McAllister-Lucas L.M., Lucas P.C., Bailey N.G., Hogaboam C.M., Lim M.S., Elenitoba-Johnson K.S. (2015). Conversion of the LIMA1 tumour suppressor into an oncogenic LMO-like protein by API2-MALT1 in MALT lymphoma. Nat. Commun..

[83] Damacharla D., Thamilselvan V., Zhang X., Mestareehi A., Yi Z., Kowluru A. (2019). Quantitative proteomics reveals novel interaction partners of Rac1 in pancreatic beta-cells: Evidence for increased interaction with Rac1 under hyperglycemic conditions. Mol. Cell. Endocrinol..

[84] Katoh M., Igarashi M., Fukuda H., Nakagama H., Katoh M. (2013). Cancer genetics and genomics of human FOX family genes. Cancer Lett..

[85] Senyilmaz D., Virtue S., Xu X., Tan C.Y., Griffin J.L., Miller A.K., Vidal-Puig A., Teleman A.A. (2015). Regulation of mitochondrial morphology and function by stearoylation of TFR1. Nature.

[86] O’Donnell K.A., Yu D., Zeller K.I., Kim J.W., Racke F., Thomas-Tikhonenko A., Dang C.V. (2006). Activation of transferrin receptor 1 by c-Myc enhances cellular proliferation and tumorigenesis. Mol. Cell. Biol..

[87] Wu J., Minikes A.M., Gao M., Bian H., Li Y., Stockwell B.R., Chen Z.N., Jiang X. (2019). Intercellular interaction dictates cancer cell ferroptosis via NF2-YAP signalling. Nature.

[88] Lu D., Yang Z., Xia Q., Gao S., Sun S., Luo X., Li Z., Zhang X., Li X. (2020). ACADSB regulates ferroptosis and affects the migration, invasion, and proliferation of colorectal cancer cells. Cell Biol. Int..

[89] Sukma Dewi I., Celik S., Karlsson A., Hollander Z., Lam K., McManus J.W., Tebbutt S., Ng R., Keown P., McMaster R. (2017). Exosomal miR-142-3p is increased during cardiac allograft rejection and augments vascular permeability through down-regulation of endothelial RAB11FIP2 expression. Cardiovasc. Res..

[90] Guay C., Kruit J.K., Rome S., Menoud V., Mulder N.L., Jurdzinski A., Mancarella F., Sebastiani G., Donda A., Gonzalez B.J. (2019). Lymphocyte-Derived Exosomal MicroRNAs Promote Pancreatic beta Cell Death and May Contribute to Type 1 Diabetes Development. Cell Metab..

[91] Cortes-Troncoso J., Jang S.I., Perez P., Hidalgo J., Ikeuchi T., Greenwell-Wild T., Warner B.M., Moutsopoulos N.M., Alevizos I. (2020). T cell exosome-derived miR-142-3p impairs glandular cell function in Sjogren’s syndrome. JCI Insight.

[92] Guiot J., Cambier M., Boeckx A., Henket M., Nivelles O., Gester F., Louis E., Malaise M., Dequiedt F., Louis R. (2020). Macrophage-derived exosomes attenuate fibrosis in airway epithelial cells through delivery of antifibrotic miR-142-3p. Thorax.

[93] Cai L., Chao G., Li W., Zhu J., Li F., Qi B., Wei Y., Chen S., Zhou G., Lu X. (2020). Activated CD4(+) T cells-derived exosomal miR-142-3p boosts post-ischemic ventricular remodeling by activating myofibroblast. Aging (Albany NY).

[94] Zhou C., Zhang Y., Yan R., Huang L., Mellor A.L., Yang Y., Chen X., Wei W., Wu X., Yu L. (2021). Exosome-derived miR-142-5p remodels lymphatic vessels and induces IDO to promote immune privilege in the tumour microenvironment. Cell Death Differ..

[95] Perez-Riverol Y., Bai J., Bandla C., Garcia-Seisdedos D., Hewapathirana S., Kamatchinathan S., Kundu D.J., Prakash A., Frericks-Zipper A., Eisenacher M. (2022). The PRIDE database resources in 2022: A hub for mass spectrometry-based proteomics evidences. Nucleic Acids Res..

