# Supplementary material for: Changes of Protein Expression after CRISPR/Cas9 Knockout of miRNA-142 in Cell Lines Derived from Diffuse Large B-Cell Lymphoma
Source: Cancers (Basel). 2022 Oct 14;14(20):5031. doi: 10.3390/cancers14205031 (PMC9600116; doi:10.3390/cancers14205031)

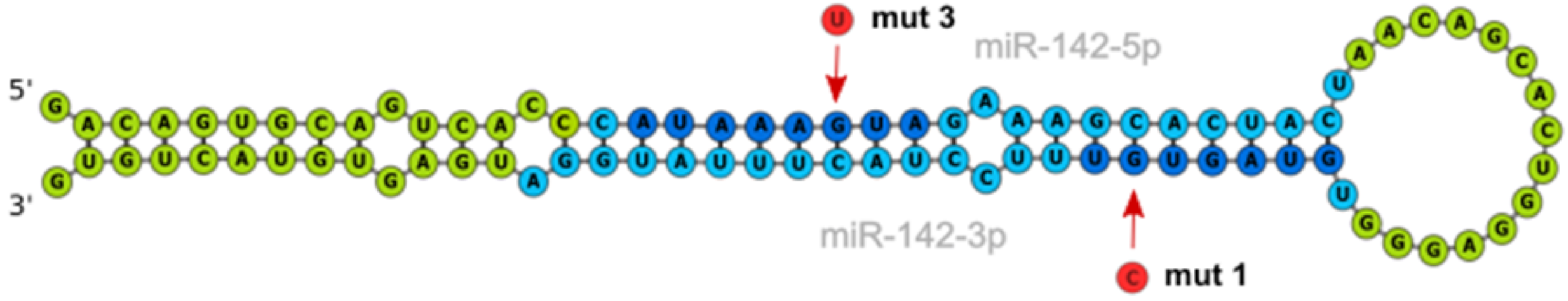

Supplementary Figure S1: Point mutations M1 and M3 in miR-142. Shown is the secondary structure of pri-miR-142. Mutations M1 and M3 occurring in DLBCL tumors studied in this work are indicated in red and annotated as mut 1 and 3. Mut 1 (U>C) is found in the seed region of miR-142-3p. Mut 3 (G>U) is in the seed of miR-142-5p. The mature miRNA species are shown in blue and the seed sequences in dark blue. The secondary structure was generated by using the RNAfold web server of the University of Vienna: (http://rna.tbi.univie.ac.at/cgi-bin/RNAWebSuite/RNAfold.cgi).

A

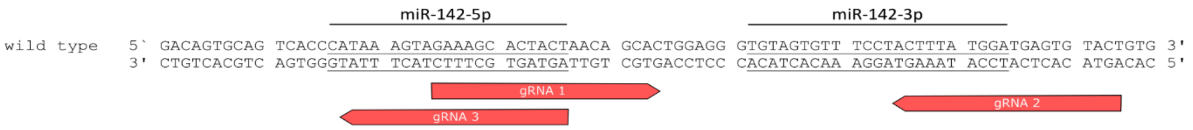

B

name	sequence (5‘-3‘)	strand	GC-content (%)
gRNA 1	GAAAGCACTACTAACAGCAC	+	48
gRNA 2	AGTACACTCATCCATAAAGT	-	39
gRNA 3	AGTAGTGCTTTCTACTTTAT	-	39

Supplementary Figure S2. (A) Genomic sequence of pri-miR-142. The positions of the mature miR-142-3p and -5p are underlined. The positions of the gRNAs are shown as red arrows. (B) Sequences of the three gRNAs used.

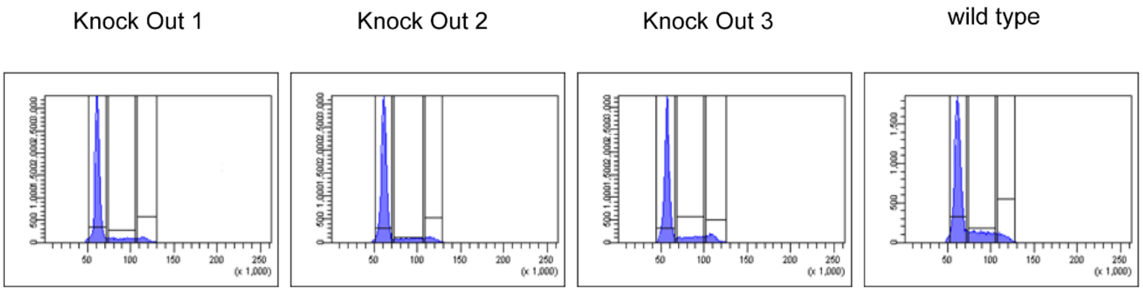

Supplementary Figure S3. Cell cycle analysis of the three BJAB knockout cell lines vs. BJAB-wild type cells. Cell cycle analysis was carried out by FACS analysis. Cells were stained with propidium iodide. The relative amounts of cells in G1, S and G2/M are shown in Figure 2B.

Supplementary Figure S4

WB PKN2 uncropped

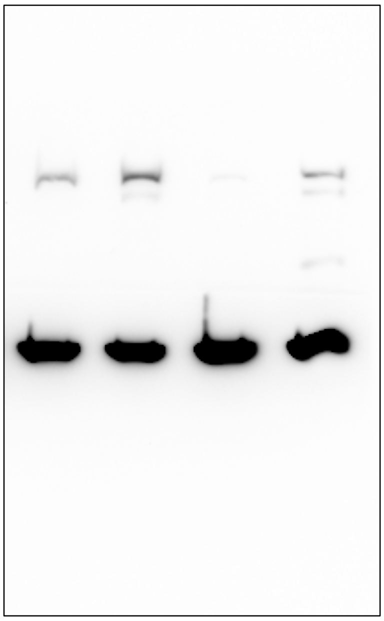

WB EZRIN uncropped

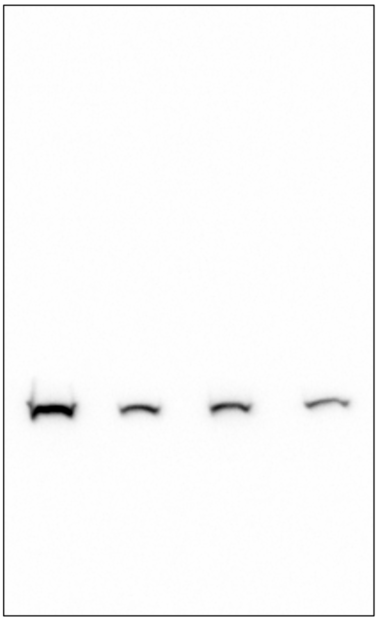

WB GAPDH uncropped

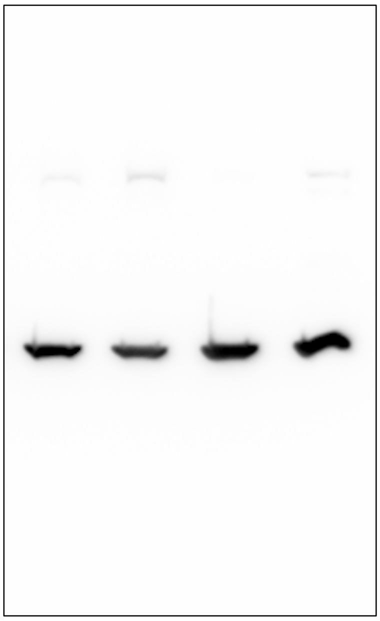

Supplement: Supplementary file 1 [file cancers-14-05031-s001.zip › Supplementary Figures S1-S4.pdf]
